# Does Metamizole Cause Less Acute Kidney Injury than Non-Steroidal Anti-Inflammatory Drugs When Combined with Diuretics and Antihypertensives?

**DOI:** 10.3390/toxics13050417

**Published:** 2025-05-21

**Authors:** Dulce Maria Calvo, Luis Carlos Saiz, Leire Leache, Maria C. Celaya, Marta Gutiérrez-Valencia, Alvaro Alonso, Juan Erviti

**Affiliations:** 1 PhD Program, Autonomous University of Barcelona (UAB), 08193 Barcelona, Spain; 2 Innovation and Organization Unit, Navarre Health Service, 31008 Pamplona, Spain; lc.saiz.fernandez@navarra.es (L.C.S.); marta.gutierrez.valencia@navarra.es (M.G.-V.); juan.erviti.lopez@navarra.es (J.E.); 3 Navarra Health Research Institute (IdiSNA), 31008 Pamplona, Spain; leire.leache.alegria@navarra.es (L.L.); mccelaya.lecea@navarra.es (M.C.C.); 4 Pharmacy and Services Sub-Directorate, Navarre Health Service, 31008 Pamplona, Spain; 5 Department of Epidemiology, Rollins School of Public Health, Emory University, Atlanta, GA 30322, USA; alvaro.alonso@emory.edu

**Keywords:** acute kidney injury, antihypertensives, diuretics, metamizole, non-steroidal anti-inflammatory agents, safety, triple whammy

## Abstract

The concurrent use of (a) diuretics, (b) renin–angiotensin–aldosterone system inhibitors (RAASIs), and (c) non-steroidal anti-inflammatory drugs (NSAIDs) or metamizole, known as the triple whammy (TW) combination, increases the risk of acute kidney injury (AKI). This study compared TWs including metamizole versus NSAIDs regarding hospitalisation for AKI, need for renal replacement therapy (RRT), and all-cause mortality during hospitalisation. Serum creatinine (sCr) and estimated glomerular filtration rate (eGFR) changes in the first year after TW initiation were also assessed. A nested case–control study was conducted within a cohort of adults receiving TW therapy (2009–2018). Logistic regression models analysed the associations between TW type and outcomes. Among 65,077 individuals (mean age 79.7 years; 26.3% male), TW including an NSAID was associated with a lower risk of AKI-related hospitalisation [adjusted odds ratio (aOR) 0.81, 95%CI 0.74–0.87] and all-cause mortality (aOR 0.64, 95%CI 0.49–0.82) compared to TW including metamizole. No significant differences were found in other variables. These findings suggest that TW including an NSAID may reduce the risk of AKI-related hospitalisation and mortality compared to TW including metamizole, although kidney function parameters remained unaffected. Further research is needed to confirm these results.

## 1. Introduction

The term “triple whammy” (TW) corresponds to the concurrent use of diuretics, renin–angiotensin–aldosterone system inhibitors (RAASIs), and non-steroidal anti-inflammatory drugs (NSAIDs) [1]. This combination of drugs has been linked to an elevated risk of acute kidney injury (AKI) [2,3,4,5]. Metamizole is one of the most prescribed analgesics in Spain, with more than 11% of the total population receiving a prescription in 2021 [6]. Currently, it is marketed in 18 EU countries. VigiAccess, a global database maintained by the World Health Organization (WHO), has registered 38,773 reports of suspected side effects associated with metamizole through May 2024 [7]. Of these, 728 (1%) involved kidney and urine system injury, of which 245 corresponded to acute renal failure and 187 to related diagnoses. Most of these reports were registered in Europe (46%). Up to May 2024, 14,592 suspected side-effect reports for metamizole have been registered in the European database of suspected adverse drug reaction reports (Eudravigilance) [8]. Spain accounts for 22.7% of these reports. A total of 696 of these reports involved the renal and urinary systems. Acute renal failure accounted for 308 notifications, of which 12.6% were fatal, not recovered, or recovered with sequelae. A rare but major side effect associated with metamizole is agranulocytosis [9].

Metamizole is a non-opioid analgesic and antipyretic, distinct from classic NSAIDs like ibuprofen and diclofenac. Its exact mechanism of action remains unclear. Unlike NSAIDs, which strongly inhibit cyclooxygenase (COX) enzymes, metamizole has a weaker and possibly indirect effect. It is considered to be a prodrug, and its active metabolites, 4-methylaminoantipyrine (MAA) and 4-aminoantipyrine (AA), stimulate the cannabinoid type 1 (CB1) and type 2 (CB2) receptors, in addition to inhibiting COX-1 and COX-2. This enhances the activity of the descending pain-inhibitory pathway and suggests a dual role in the modulation of pain and inflammation. Inhibition of COX-3 in the central nervous system, as well as activation of the endogenous opioid system, has also been proposed. Since prostaglandins are crucial for renal perfusion, especially during stress (e.g., dehydration, heart failure, or chronic kidney disease), NSAIDs can cause complications such as acute kidney injury, interstitial nephritis, or worsening of pre-existing kidney disease. By preserving the prostaglandin pathways more than NSAIDs, metamizole appears to be less nephrotoxic and is often preferred in patients at risk of renal damage [10].

To date, there is not enough evidence to support the notion that metamizole is associated with a lower risk of AKI compared to NSAIDs. Additionally, no studies have assessed the likelihood of developing AKI when metamizole is combined with diuretics and RAASIs.

A published study reported a higher risk of hospitalisation due to AKI as a secondary outcome in patients exposed to a TW compared to those not exposed (adjusted odds ratio [aOR] 1.36, 95%CI 1.32–1.40). This risk was even higher when the TW included metamizole rather than an NSAID [10,11]. This result can be partially explained by a possible indication bias, as metamizole is often prescribed instead of NSAIDs for patients with mildly impaired kidney function or other cardiovascular risk factors. Therefore, determining the potential impact of metamizole as part of a TW on kidney function and other clinical outcomes, in comparison to TWs with NSAIDs, is crucial.

The study tried to prove that patients receiving TW therapy with metamizole have a similar risk of hospitalisation due to AKI and associated clinical consequences, as compared to a TW with NSAIDs.

The objective of this study was to analyse the impact of the TW combination that includes metamizole as compared to the TW that includes an NSAID in terms of risk of hospitalisation for AKI, kidney function parameters, and other morbi-mortality outcomes.

## 2. Materials and Methods

### 2.1. Study Design and Source of Data

The current study was carried out on the basis of a population-based case–control study nested in a cohort that analysed the risk of hospitalisation for AKI with the TW combination [9,10]. All of the data for this study were extracted from the Spanish Database for Pharmacoepidemiological Research in Public Administration (BIFAP, https://www.bifap.org), managed by the Spanish Agency for Medication and Healthcare Products and integrated in DARWIN EU [6,12]. At this moment, the database includes more than 22 million medical records [6].

### 2.2. Study Cohort and Follow-Up

The cohort consisted of adults exposed to the TW combination, namely, the simultaneous prescription of at least one drug from each of the following drug classes between 1 January 2009 and 31 December 2018 and in the 12 months prior to the index date: (a) diuretics, (b) RAASIs [angiotensin-converting enzyme inhibitors (ACEIs), angiotensin II receptor blockers (ARBs), or aliskiren], and (c) NSAIDs or metamizole (see Table S1). The date of the first prescription for any of these drugs was defined as the cohort entry date [11]. The subjects had to be at least 18 years old at cohort entry and had to have at least one year of follow-up in the BIFAP prior to cohort entry.

The index date corresponds to the date of the first hospitalisation for AKI during the follow-up for the cases. As for the matched controls, the corresponding index date was that of the matched case. A case corresponds to a patient who suffered hospitalisation due to AKI during the study period. Each case was matched with up to 10 randomly selected controls of the same age, sex, and region in Spain, and according to the calendar year of registration in the BIFAP. The controls did not suffer hospitalisation due to AKI on the index date corresponding to their case.

Exposure to TW could be at any time in the previous 12 months to the index date, and not necessarily during the entire period. If this exposure started before 1 January 2009, this date was considered to be the date of the cohort’s entry. Patients who were exposed simultaneously to both metamizole and an NSAID as part of the TW were excluded. 

Subjects were followed-up until the first hospitalisation due to AKI during the study period (1 January 2010–31 December 2018), the occurrence of cancer (except basal cell carcinoma), death from any cause, loss to follow-up, or study termination (31 December 2018), whichever occurred first. 

### 2.3. Exposure to TW and Comparison

The exposure referred to the period of time covered by drug prescriptions. The duration of a drug prescription was considered to be 30 days unless specifically stated. A treatment course of a drug was considered to be continuous when there was a gap of no more than 60 days between the last day of a prescription and the first day of a subsequent prescription of the same drug. A TW treatment was considered to be continuous when there was a gap of no more than 30 days between the last day of a TW exposure and the first day of a subsequent TW exposure [11].

In the event of patients who were exposed to a TW including metamizole and to a TW including an NSAID (or vice versa) in a sequential manner in the 12 months prior to the index date, the exposure to the TW closest in time to the index date was considered as a reference for the analyses.

The TW combination consisted of the simultaneous prescription of at least one drug from each of the following drug classes at any moment during the 12 months prior to the index date: (a) diuretics, (b) RAAS inhibitors (ACEI, ARB, or aliskiren), and (c) NSAIDs or metamizole [11]. The study compared patients exposed to the TW combination including metamizole (and not NSAIDs) vs. patients exposed to the TW combination including an NSAID (and not metamizole). Additionally, we compared both groups according to the duration of TW exposure, categorising subjects into three groups: one month, two to six months, and seven to twelve months of exposure.

### 2.4. Variables

The primary outcome was hospitalisation for AKI. The ninth and tenth revisions of the International Classification of Diseases (ICD-9 and ICD-10) were used to identify cases of hospitalisation in which AKI figured as the main or secondary diagnosis (Table S2). 

Additionally, the following secondary outcomes were assessed:

- Need for renal replacement therapy (RRT) during hospitalisation due to AKI.
- All-cause mortality during hospitalisation due to AKI.
- Changes in serum creatinine (sCr) and estimated glomerular filtration rate (eGFR) during the first 12 months after the beginning of the TW exposure, as compared to sCr and eGFR at the beginning of the TW exposure (baseline).
- Changes in sCr and eGFR from the beginning of the TW exposure (i) until day 90, (ii) from day 91 to day 180, and (iii) from day 181 to day 365.

The need for RRT and all-cause mortality outcomes were analysed among the participants who suffered hospitalisation due to AKI (cases).

The analysis of the changes in sCr and eGFR during the first 12 months after the beginning of TW exposure was performed in the patients (both cases and controls) who had sCr and/or eGFR data at baseline and at some later timepoint in the following 12 months. The sCr and eGFR at the beginning of TW exposure or the last values in the previous 12 months were considered to be the baseline data. If continuous TW exposure closest in time to the index date initiated before 1 January 2009, this date was considered as the baseline for these analyses. 

For the analysis of the changes in sCr and eGFR during the first 12 months after the beginning of the TW exposure, the last sCr and eGFR values available in the 12 months were considered as the follow-up values in the cases of patients in whom the TW exposure was continued during 12 months without interruptions, and the value closest in time to the end of the TW exposure was selected in the cases of patients in whom the TW exposure was interrupted during the 12 months. The same criterion was followed for the analysis of the changes in sCr and eGFR until day 90, from day 91 to day 180, and from day 181 to day 365 from the beginning of the TW exposure. In cases where the eGFR was not available in the BIFAP, it was calculated using the CKD-EPI (Chronic Kidney Disease Epidemiology Collaboration) equation, whenever possible [13].

Information on age, sex, body mass index (BMI) (BMI closest in time to the index date or calculated through the height and weight values closest in time to the index date), comorbidities (chronic kidney disease (CKD), cardiac and/or valvular diseases, diabetes mellitus, hypertension) registered up to the index date, and exposure to drugs of the TW combination and to other drugs that can affect kidney function in the 12 months prior to the index date was collected and considered as potential confounding variables. 

### 2.5. Data Analysis

The baseline characteristics of the whole cohort were analysed. Categorical variables were described as frequency distributions, and quantitative variables were described as the mean and standard deviation (SD). 

Results in categorical outcomes were analysed using unadjusted and adjusted models. Crude analyses of categorical outcomes were carried out using chi-squared tests or Fisher’s test, and in the case of continuous variables, Student’s *t*-test was used. Adjusted results were estimated using multivariable conditional logistic regression models. Propensity score matching was employed to control for measured confounders, utilising a calliper width equal to 0.01 of the standard deviation of the logit of the propensity score, implemented through a greedy nearest-neighbour matching algorithm with a 1:1 matching ratio.

Categorical outcomes were presented as odds ratios (ORs) and adjusted ORs with 95% confidence intervals (95%CIs). Quantitative outcomes were presented as the mean difference (MD) with SD.

Furthermore, we conducted the following sensitivity analyses of the primary outcome: 

(a) Restricting to cases and controls who had baseline eGFR or sCr data available, extracted from the BIFAP or calculated through the CKD-EPI equation. Multiple imputation by chained equations (MICE) with 5 imputations was performed to handle missing data and to reduce bias.

Since patients with a worse eGFR might be more likely to receive metamizole instead of NSAIDs, this analysis was adjusted for baseline eGFR (extracted from the BIFAP or calculated through the CKD-EPI equation), in addition to the rest of the confounding factors (except CKD), to reduce the possibility of indication bias. 

(b) Excluding cases and controls who were exposed to a TW including metamizole and to a TW including an NSAID in a sequential manner in the 12 months prior to the index date.

(c) Restricting to cases in which AKI featured as the primary hospitalisation diagnosis. 

A subgroup analysis of the primary outcome was performed in patients older than 75 years, in patients with eGFR < 60 mL/min/1.73 m^2^ at baseline, and in patients diagnosed with CKD at baseline.

## 3. Results

The cohort included 65,077 people (25,364 with a TW including metamizole, and 39,713 with a TW including an NSAID). The mean follow-up of the cohort was 72.5 (SD 43.5) months.

The baseline characteristics of the cohort are displayed in Table 1, where 38.3% were males and the mean age at baseline was 79.7 years (SD 9.15). Diabetes mellitus, CKD, and cardiac and/or valve disease were more frequent in the group with TWs including metamizole. In general, more people from the group with TWs including metamizole were exposed to nephrotoxic drugs. The sCr was significantly higher in the group with TWs including with metamizole, and the eGFR was significantly lower in this group. There were significantly more people with eGFR < 60 mL/min/1.73 m^2^ in the group with TWs including metamizole. The length of continuous and overall TW exposure was significantly longer in the group with TWs including metamizole.

Additionally, the balance of covariates between the two groups of patients exposed to the TW combination, one including metamizole and the other including an NSAID, was evaluated following propensity score matching, with 20,064 patients in each group. The evaluation revealed an acceptable balance in most covariates; however, small but statistically significant standardised differences were observed in age, comorbidities such as hypertension, eGFR < 60 mL/min/1.73 m^2^, and the duration of continuous and overall TW exposure (Table S3).

### 3.1. Outcomes in Primary and Secondary Variables

Table 2 presents the results of the primary outcome. The risk of hospitalisation for AKI was significantly lower among individuals exposed to the combination including an NSAID compared to those exposed to a TW including metamizole (aOR 0.81, 95%CI 0.74–0.87). Furthermore, the post-propensity score matching analysis provided a more precise interpretation of the associations between TW combinations and AKI, minimising confounding bias (aOR 0.87, 95%CI 0.80–0.96; *p* = 0.004) (Table S4). 

Prolonged exposure to the TW with either metamizole or NSAIDs was associated with an increased risk of hospitalisation due to AKI. The risk of hospitalisation increased gradually with longer exposure in the case of the combination with metamizole (aOR for patients with the combination from 2 to 6 months: 1.05, 95%CI 1.55–1.75; aOR for patients with the combination from 7 months to 12 months: 1.20, 95%CI 1.04–1.39). In the group of TWs with NSAIDs, only the subgroup with exposure from 7 to 12 months had an increased risk of hospitalisation (aOR 1.08, 95%CI, 0.93–1.26) (Table S5).

All-cause mortality during hospitalisation for AKI was also significantly lower with TWs including an NSAID (aOR 0.64, 95%CI 0.49–0.82) (Table 3). There were no statistically significant differences between groups in the requirement of RRT during hospitalisation (Table 3). 

As for the kidney function parameters, there were no significant differences between TW including metamizole and TW including NSAIDs in the changes in sCr or in eGFR, except for the change in eGFR within 90 days from baseline. In this case, there was an increase in eGFR in the group with TW including metamizole, and there was a decrease in the group with TW including NSAIDs (MD −0.9; SD 10.7; *p* value = 0.003).

### 3.2. Sensitivity Analysis of the Primary Outcome

The results of the sensitivity analysis of hospitalisation due to AKI were coincident with those from the primary analysis. When restricting the analyses of the primary outcome to patients with baseline eGFR or sCr data available (3725 cases and 24,899 controls), the adjusted risk of hospitalisation due to AKI was significantly lower in patients exposed to a TW including NSAIDs, as compared to those exposed to a TW including metamizole (aOR 0.78, 95%CI 0.66–0.93) (Table S6, Figure S1). A similar result was observed after applying multiple imputations for missing eGFR and sCr data from 36.453 (aOR 0.83; 95%CI 0.70–0.98).

When excluding from the analysis cases and controls exposed to TWs including metamizole and to TWs including an NSAID in a sequential manner, or vice versa, the group with TWs including an NSAID was also associated with a significantly lower risk of hospitalisation due to AKI (aOR 0.79, 95%CI, 0.72–0.87) (Table S6, Figure S1).

We did not find a significant difference between groups in the risk of hospitalisation due to AKI when the analysis was restricted to cases where AKI featured as the primary hospitalisation diagnosis (aOR 0.88, 95%CI, 0.6–1.12) (Table S6, Figure S1).

### 3.3. Subgroup Analysis of the Primary Outcome

When restricting the analysis of hospitalisation due to AKI to patients older than 75 years (6286 cases and 42,060 controls), the risk was significantly lower in the group with TWs including an NSAID (aOR 0.83, 95%CI 0.76–0.91), coinciding with the primary analysis (Table S6, Figure S1). However, in the subgroup of patients with eGFR < 60 mL/min/1.73m^2^, with aOR 0.97 (95%CI, 0.71, 1.33) or CKD at baseline (aOR 1.03, 95%CI, 0.56, 1.92), there was no significant difference between groups (Table S6, Figure S1).

## 4. Discussion

This study, comparing the exposure to TWs including metamizole and TWs including an NSAID in terms of the risk of hospitalisation due to AKI and other relevant morbi-mortality outcomes, is so far one of the first to bring previously unknown evidence based on a quality study with a substantial sample size. The cohort consisted of 65,077 Spanish people, with a mean follow-up of six years. According to our data, patients exposed to TWs including NSAIDs would have a significantly lower risk of hospitalisation due to AKI than those with TWs including metamizole, including the subgroup of patients older than 75 years. These findings were confirmed when restricting the analysis to patients with baseline kidney function parameters available, and when excluding patients who had received both types of TW combinations in a sequential way. However, we found no significant differences when restricting to those patients in whom AKI was the primary hospitalisation diagnosis, nor when restricting to people with CKD or eGFR < 60 mL/min/1.73 m^2^ at baseline. The subgroup analysis in patients with eGFR < 60 mL/min/1.73 m^2^ at baseline and in those diagnosed with CKD at baseline did not show significant differences between groups. These results could be affected by residual confounding factors.

Regarding outcomes related to kidney function, such as requirement of RRT, and changes in sCr or eGFR, there were no significant differences between the groups in general. We also found higher all-cause mortality during hospitalisation for AKI in the group with TWs including metamizole. A possible explanation for the higher risk of hospitalisation due to AKI and all-cause mortality observed with TWs including metamizole could be related to a potential indication bias, as metamizole is often prescribed instead of NSAIDs in patients with somewhat impaired kidney function, other cardiovascular risk factors, or worse clinical condition in general. This is consistent with the fact that the baseline characteristics of patients receiving a TW, including those taking metamizole, generally showed a higher prevalence of comorbidities such as impaired kidney function, CKD, cardiovascular disease, and diabetes, and they were exposed to nephrotoxic drugs to a greater extent. Additionally, the patients treated with a TW including metamizole were significantly older. Moreover, metamizole users were slightly more frequently monitored, implying a detection bias. These factors may have influenced the results observed. Although we adjusted for the most relevant confounding factors, this approach may have been insufficient to fully balance the baseline differences, and residual confounding may still exist.

### 4.1. Evidence from Other Published Studies

Published data indicate that metamizole has the potential to cause nephrotoxicity. A study by Canillas et al. [14] found that the use of metamizole after major orthopaedic surgery was significantly associated with the development of AKI in patients with advanced chronic liver disease (aOR 7.0; 95%CI 1.8–27.2). 

Several studies have been published analysing the risk of AKI with the use of the TW combinations, many of them retrospective observational studies [3,14,15,16,17,18], which showed that the use of TW increases the risk of AKI. However, we found no studies assessing the risk of hospitalisation due to AKI and other clinically relevant outcomes with the TW combination including metamizole as compared to the TW combinations including an NSAID.

Our findings, along with those from the literature on the subject, do not demonstrate that TWs including metamizole may reduce kidney-related injury compared to TWs including NSAIDs. Moreover, our results favour the use of NSAIDs, although, given the potential biases due to the observational nature of this study, the findings should be confirmed in further studies. 

### 4.2. Strengths

To the best of our knowledge, this is the first study aimed at addressing the real-world clinical impact of metamizole as part of a TW combination, in terms of kidney function and other relevant morbi-morbidity outcomes, based on a large and robust, validated, real-world clinical database. Considering the widespread use of metamizole in certain countries, and especially its use as an alternative to NSAIDs, the findings of this study may have important implications for routine clinical practice.

One of the most notable strengths of this study is the population-based nature of the BIFAP database through which the study was conducted. This database collates over 40% of Spain’s total population [6]. and is validated for the study outcomes. Also, this database aims to help clinicians’ decision-making about drugs, for improving their use in clinical practice. Furthermore, the large sample size of the cohort, the case validation procedure, the adjustment of the results by the most relevant confounding factors, and the subgroup and sensitivity analyses carried out produced reliable results and ensured the validity of the conclusions drawn. Furthermore, the use of statistical techniques, such as propensity score matching to reduce selection and confounding bias, as well as multiple imputations to address bias from missing data, further strengthens the robustness of this study.

A validation process was conducted to identify true cases. Additionally, the likelihood of failing to identify other true cases using ICD-9/10 codes was minimal, as the CMBD is a reliable source where hospitalisation diagnoses are recorded at discharge and verified by qualified professionals before being integrated into information systems. Nevertheless, the possibility of missing other relevant codes was low. Moreover, we also used ICPC-2 (U99.01 for CKD) in addition to ICD-9 and ICD-10.

Another strength of this study was the availability of information on laboratory parameters such as sCr and eGFR. Increased sCr, impaired eGFR, and/or abnormal urine results are typically the first signs of kidney disease. So far, there are no studies analysing the impact of the TW on kidney function parameters. In our study, we did not observe relevant differences between TWs including metamizole and TWs including an NSAID in this respect.

### 4.3. Limitations

A potential indication bias may exist, since practitioners are more likely to prescribe metamizole over NSAIDs to people who have a higher risk of cardiovascular disease or impaired renal function. In order to reduce this possible bias, we conducted multivariable conditional logistic regression models of the outcomes, adjusting for the most relevant confounding factors.

We considered factors like age, other health conditions, and other harmful drugs in our analyses. However, we acknowledge that there may still be unmeasured factors that could have caused some confusion in the results due to the observational nature of this study. Specifically, frailty status, which was not explicitly recorded in our dataset, could have influenced both the likelihood of receiving metamizole instead of NSAIDs and the observed outcomes, given the preferential use of metamizole in more vulnerable populations. Comparably, the use of over-the-counter (OTC) NSAIDs, which was not routinely documented, may have led to misclassification of exposure, which could bias the results. Considering that an unmeasured confounder such as frailty is strongly associated with both the exposure and the outcome, the observed associations would be attenuated if frailty were distributed in an unbalanced way between groups. However, given the magnitude of the adjusted odds ratios, a strong unmeasured confounder would be required to fully account for the results. Future studies should include direct measures of frailty and systematic recording of OTC NSAID use, like other confounding factors, to validate these findings.

Baseline kidney function data (eGFR, sCr) were missing for some patients, which could have introduced selection bias. Nevertheless, sensibility analysis was performed with cases who had baseline eGFR or sCr data available, and the results were consistent with the primary outcome. Also, baseline eGFR was used as a confounding factor (except CKD) to reduce the possibility of indication bias.

The choice of hospitalisation for AKI as the primary outcome carries the risk of excluding outpatient AKI events, potentially underestimating the overall risk. However, for us, hospitalisation was a relevant primary outcome and was obtained from discharge diagnoses in the CMBD. In addition to the availability of these data, hospitalisation for AKI in our study was considered at any time within the 12 months prior to the index date. Despite this, differentiating between community-acquired and hospital-acquired AKI was not an objective of this study. The latter has been more extensively studied in high-budget countries, whereas the former is thought to occur under conditions such as dehydration and infectious processes.

NSAIDs can be obtained from community pharmacies without a physician’s prescription. Information regarding the consumption of NSAIDs without a prescription is not registered in the BIFAP; thus, this issue may have impacted the results in some way. 

Growing polypharmacy because of comorbidities might make selecting appropriate analgesics much more challenging due to the increased possibility of interactions that could have negative consequences, especially when prescribing NSAIDs. For example, the concurrent use of RAASIs and diuretics is prevalent in managing conditions like hypertension and heart failure. NSAIDs and metamizole are widely accessible and are sometimes used without medical supervision. This leads to frequent exposure to the TW combination with NSAIDs or metamizole, which has been associated with a significantly increased risk of AKI. Particular attention should be given to patients with advanced age, dehydration, CKD, hypertension, cardiovascular diseases, arteriosclerosis, high salt intake, or those using other nephrotoxic drugs, as these factors increase susceptibility to AKI.

While metamizole has been considered to have a more favourable renal profile compared to traditional NSAIDs, there is currently insufficient evidence to support this, and recent studies have highlighted potential nephrotoxic effects, especially in high-risk populations [19]. Therefore, information regarding the careful use of metamizole, instead of NSAIDs, should be shared with practitioners. Moreover, regular monitoring of kidney function in patients with these risk factors should be established as a clinical practice, with a reassessment of the necessity of continuing all three medications concurrently. Additionally, educating patients about avoiding metamizole’s use without prescription, especially when on RAASIs or diuretics, is very important.

## 5. Conclusions

Individuals exposed to TWs including NSAIDs had a considerably lower risk of hospitalisation for AKI and all-cause mortality during hospitalisation than those patients exposed to TWs including metamizole. However, there were no statistically significant differences between the two groups in the changes in sCr or eGFR, nor in terms of RRT requirement during hospitalisation. Further research is warranted to confirm these results and to better understand the deleterious effects of metamizole.

## Figures and Tables

**Table 1:** Baseline characteristics of the cohort.

Variable	TW Including Metamizole	TW Including NSAIDs	*p*
**N**	**25,364 **	**39,713 **	
**Age** (years), mean (SD)	81.7 (8.6)	77.8 (9.7)	<0.0001
**Sex**, n males (%)	7930 (31.3%)	17,027 (42.9%)	<0.0001
**Body mass index** (kg/m^2^), n (%)			<0.0001
<20.0	124 (0.5%)	116 (0.3%)	
≥20.0 to <25.0	1550 (6.1%)	2124 (5.4%)	
≥25.0 to <30.0	4239 (16.7%)	7461 (18.8%)	
≥30.0 to <35.0	3597 (14.2%)	6075 (15.3%)	
≥35.0	523 (2.1%)	719 (1.8%)	
Not available	13,937 (55.0%)	20,960 (52.8%)	
**Follow-up** (months), mean (SD)	92.7 (49.1)	70.5 (38.0)	<0.0001
**Comorbidities, n (%)**			
CKD	3330 (13.1%)	2664 (6.7%)	<0.0001
Cardiac and/or valvular diseases ^a^	10,381 (40.9%)	10,702 (27.0%)	<0.0001
Diabetes	8325 (32.9%)	10,804 (27.2%)	<0.0001
Hypertension	21,188 (83.5%)	34,618 (87.2%)	<0.0001
sCr (mg/dL), mean (SD)	1.04 (0.41)	0.99 (0.33)	<0.0001
eGFR (mL/min/1.73 m^2^), mean (SD)	63.5 (22.2)	70.3 (23.4)	<0.0001
eGFR KDIGO categories ^b^			<0.0001
Grade 1 (≥90 ml/min/1.73 m^2^)	1155 (9.5%)	2940 (17.8%)	
Grade 2 (≥60 to <90 mL/min/1.73 m^2^)	5478 (45.1%)	7888 (47.8%)	
Grade 3 (≥30 to <60 mL/min/1.73 m^2^)	4834 (39.8%)	5209 (31.6%)	
Grade 4 (≥15 to <30 mL/min/1.73 m^2^)	618 (5.1%)	398 (2.4%)	
Grade 5 (<15 mL/min/1.73 m^2^)	51 (0.4%)	53 (0.3%)	
**Drug exposure in the 12 months prior to the index date,** n (%)			
α-Blockers	1112 (4.4%)	1352 (3.4%)	<0.0001
β-Blockers	5270 (20.8%)	6026 (15.2%)	<0.0001
Calcium channel blockers	7413 (29.2%)	9911 (25.0%)	<0.0001
Oral antiplatelet agents	7440 (29.3%)	9890 (24.9%)	<0.0001
Anticoagulants	4941 (19.5%)	4062 (10.2%)	<0.0001
Digoxin	958 (3.8%)	797 (2.0%)	<0.0001
Antianginal and antiarrhythmic drugs	3448 (13.6%)	3615 (9.1%)	<0.0001
Statins	10,653 (42.0%)	14,894 (37.5%)	<0.0001
Antidiabetic drugs	6380 (25.2%)	8461 (21.3%)	<0.0001
Acyclovir	47 (0.2%)	47 (0.1%)	0.03
Beta-lactams	3508 (13.8%)	5154 (13.0%)	0.002
Quinolones	2260 (8.9%)	3023 (7.6%)	<0.0001
Sulphonamides	172 (0.7%)	146 (0.4%)	<0.0001
Rifampicin	25 (0.1%)	23 (0.1%)	0.09
Immunosuppressants	130 (0.5%)	148 (0.4%)	0.008
Acetaminophen	11,933 (47.1%)	15,236 (38.4%)	<0.0001
Systemic corticosteroids	1800 (7.1%)	2456 (6.2%)	<0.0001
Bisphosphonates	1214 (4.8%)	2005 (5.1%)	0.13
Allopurinol	2217 (8.7%)	3785 (9.5%)	0.0007
Penicillamin	0 (0%)	2 (0.01%)	0.52
Lithium	7 (0.03%)	22 (0.1%)	0.10
Nitrogen mustards	3 (0.01%)	1 (<0.01%)	0.31
Quinolines	46 (0.2%)	89 (0.2%)	0.24
Gold preparations	0 (0%)	0 (0%)	1.00
**Length of continuous TW exposure ^c^ (months),** mean (SD)	4.8 (3.8)	3.9 (3.6)	<0.0001
**Length of overall TW exposure (months),** mean (SD)	5.1 (3.8)	4.2 (3.6)	<0.0001

CKD: chronic kidney disease; eGFR: estimated glomerular filtration rate; sCr: serum creatinine. ^a^. Heart failure, ischemic cardiopathy (including acute coronary syndrome, acute myocardial infarction, angor pectoris), cardiac valvulopathy, arrhythmia, atrial fibrillation and/or auricular flutter, and congenital cardiovascular disorders. ^b^. Available for n = 12,136 from the group with TWs including metamizole, and n = 16,448 from the group with TWs including NSAIDs. ^c^. Continuous TW: when there was a gap of no more than 30 days between the last day of a TW exposure and the first day of a subsequent TW exposure.

**Table 2:** Risk of hospitalisation due to acute kidney injury (AKI).

	Cases, n (%)	Controls, n (%)	OR (95%CI)	Adjusted OR ^a^ (95%CI)
**TW including metamizole and not NSAID**	4123 (49.0%)	21,241 (37.5%)	1 (Ref.)	1 (Ref.)
**TW including NSAID and not metamizole**	4299 (51.0%)	35,414 (62.5%)	0.58 (0.55, 0.63)	0.81 (0.74, 0.87)

NSAID: non-steroidal anti-inflammatory drug; OR: odds ratio; TW: triple whammy; 95%CI: 95% confidence interval. ^a^ Adjusted for comorbidities registered up to the index date (chronic kidney disease, cardiovascular and/or valvular disease, diabetes, hypertension, cerebrovascular diseases, chronic liver disease, chronic obstructive pulmonary disease, alcohol dependence, smoking, substance abuse), body mass index category, and exposure to the following drugs in the 12 months prior to the index date: α-blockers, β-blockers, calcium channel blockers, oral antiplatelet agents, anticoagulants, digoxin, antianginal, antiarrhythmic drugs, statins, antidiabetic drugs, acyclovir, beta-lactams, quinolones, sulphonamides, rifampicin, immunosuppressants, acetaminophen, systemic corticosteroids, bisphosphonates, allopurinol.

**Table 3:** Requirement of renal replacement therapy (RRT) and all-cause mortality.

	Requirement of RRT During Hospitalisation for AKI		OR (95%CI)	Adjusted OR ^a^ (95%CI)
	No, n (%)	Yes, n (%)		
**TW including metamizole and not NSAIDs**	3984 (96.6%)	139 (3.4%)	1 (Ref.)	1 (Ref.)
**TW including NSAIDs and not metamizole**	4090 (95.1%)	209 (4.9%)	1.47 (1.18, 1.82)	1.13 (0.90, 1.43)
	**All-Cause Mortality during Hospitalisation due to AKI**			
	**No, n (%)**	**Yes, n (%)**		
**TW including metamizole and not NSAIDs**	3949 (95.8%)	174 (4.2%)	1 (Ref.)	1 (Ref.)
**TW including NSAIDs and not metamizole**	4199 (97.7%)	100 (2.3%)	0.54 (0.42, 0.69)	0.64 (0.49, 0.82)

AKI: acute kidney injury; NSAID: non-steroidal anti-inflammatory drug; OR: odds ratio; TW: triple whammy; 95%CI: 95% confidence interval. ^a^. Adjusted for age, sex, comorbidities registered up to the index date (chronic kidney disease, cardiovascular and/or valvular disease, diabetes, hypertension, cerebrovascular diseases, chronic liver disease, chronic obstructive pulmonary disease, alcohol dependence, smoking), body mass index category, and exposure to the following drugs in the 12 months prior to the index date: α-blockers, β-blockers, calcium channel blockers, oral antiplatelet agents, anticoagulants, digoxin, antianginal/antiarrhythmic drugs, statins, antidiabetic drugs, beta-lactams, quinolones, sulphonamides, immunosuppressants, acetaminophen, systemic corticosteroids, bisphosphonates, allopurinol.

## Data Availability

Study databases will be provided upon formal request.

## Supplementary Materials

The following supporting information can be downloaded at https://www.mdpi.com/article/10.3390/toxics13050417/s1: Table S1: Diagnostic codes for acute kidney injury (AKI), chronic kidney disease (CKD), and renal replacement therapy (RRT). Table S2: The Anatomical Therapeutic Chemical (ATC) codes of the components of the triple whammy (TW) combination. Table S3: Baseline characteristics of the groups after propensity score matching. Table S4: Risk of hospitalisation due to acute kidney injury (AKI) after propensity score matching. Table S5: Risk of hospitalisation due to acute kidney injury (AKI) according to exposure time. Table S6: Sensitivity and subgroup analysis. Table S7: Risk of hospitalisation due to AKI restricting to cases and controls with eGFR or sCr data at baseline (at the beginning or in the 12 months previous to the beginning of the follow-up) (sensitivity analyses) with multiple imputations. Figure S1. Forest plot for sensitivity and subgroup analyses of acute kidney injury (AKI).

## Abbreviations

The following abbreviations are used in this manuscript:

AKI	Acute kidney injury
RAASIs	Renin–angiotensin–aldosterone system inhibitors
NSAIDs	Non-steroidal anti-inflammatory drugs
TW	Triple whammy
RRT	Renal replacement therapy
sCr	Serum creatinine
eGFR	Estimated glomerular filtration rate
WHO	World Health Organization
BIFAP	Pharmacoepidemiological Research in Public Administration
ACEIs	Angiotensin-converting enzyme inhibitors
ARBs	Angiotensin II receptor blockers
ICD	International Classification of Diseases
CKD-EPI	Chronic Kidney Disease Epidemiology Collaboration
BMI	Body mass index
CKD	Chronic kidney disease
SD	Standard deviation
OR	Odds ratio
CI	Confidence interval
PSM	Propensity score matching
