# Supplementary material for: Does Metamizole Cause Less Acute Kidney Injury than Non-Steroidal Anti-Inflammatory Drugs When Combined with Diuretics and Antihypertensives?
Source: Toxics. 2025 May 21;13(5):417. doi: 10.3390/toxics13050417 (PMC12115828; doi:10.3390/toxics13050417)
Supplement: Supplementary file 1 [file toxics-13-00417-s001.zip › toxics-3587954-supplementary.pdf]

Supplementary material

Table S1. Diagnostic codes for acute kidney injury (AKI), chronic kidney disease (CKD), and renal replacement therapy (RRT)

Diagnosis	Classification	Code	Description
**AKI**	**ICD-9**	584	Acute kidney injury
		584.5	Acute kidney injury with tubular necrosis
		584.6	Acute kidney injury with (papilar) necrosis of the renal cortex
		584.7	Acute kidney injury with renal medullary necrosis
		548.8	Acute kidney injury with other specified renal injury
		548.9	Acute kidney injury, unspecified
	**ICD-10**	N17.0	Acute kidney injury with tubular necrosis
		N17.1	Acute kidney injury with acute cortical necrosis
		N17.2	Acute kidney injury with medullary necrosis
		N17.8	Other acute renal failure
		N17.9	Acute kidney injury, unspecified
**CKD**	**ICPC-2**	**U99.01**	CKD
	**ICD-9**	**39.27**	Arteriovenostomy for dialysis
	**ICD-10**	**N18**	CKD
**RRT**	**ICD-9**	38.95, 39.27, 39.42, 39.43, 39.95, 39.95, 39.95, 458.21, 54.98, 792.5, 996.56, 996.68, 996.73, E870.2, E872.2, E874.2, E879.1, V45.1, V45.11, V45.12, V56, V56.0, V56.1, V56.2, V56.3, V56.31, V56.32, V56.8	
	**ICD-10**	03PY07Z, 03PY0JZ, 03PY0KZ, 03PY37Z, 03PY3JZ, 03PY3KZ, 03PY47Z, 03PY4JZ, 03PY4KZ, 3E1M39Z, 5A1D00Z, 5A1D60Z, I95.3, R88.0, T82.818A, T82.828A, T82.838A, T82.848A, T82.858A, T82.868A, T82.898A, T85.611A, T85.621A, T85.631A, T85.691A, T85.71XA, Y62.2, Y84.1, Z49.01, Z49.02, Z49.31, Z49.32, Z91.15, Z99.2	

AKI: acute kidney injury; CDK: chronic kidney disease; ICD: International Classification of Diseases; ICPC: International Classification of Primary Care; RRT: renal replacement therapy

Table S2. The Anatomical Therapeutic Chemical (ATC) codes of the components of the triple whammy (TW) combination

Drug or drug class	ATC code
**Diuretic of any class**	B05BC, C02L, C03, C07B, C07C, C07D, C08G, C09BA, C09DA, C09DX01, C09DX03, C09XA52, C09XA54, C09BX01, C09BX03, C10BX13, S01EC01, S01EC02
**Antihypertensives that inhibit the renin-angiotensin-aldosterone system (ACEI, ARB or aliskiren)**	C09, C10BX04, C10BX06, C10BX13, C10BX14, C10BX15
**NSAIDs**	A03DB04, M01AA, M01AB, M01AC, M01AE, M01AG, M01AH, M01AX01, M01AX02, M01AX04, M01AX07, M01AX13, M01AX14, M01AX17, M01AX18, M01AX22, M01AX23, M01AX24, M01AX26, M01AX68, M01AX74, M01B
**Metamizole**	N02BB02, A03DB04

ACEI: Angiotensin Converting Enzyme Inhibitors; ARB: Angiotensin II Receptor Blockers; NSAIDs: non-steroidal anti-inflammatory drugs

Table S3. Baseline characteristics of the groups after propensity score matching

Variable	TW including metamizole	TW including NSAID	p
**N**	**20,064**	**20,064**	
**Age** (years), mean (SD)	80.8 (8.8)	80.5 (8.3)	0.004
**Sex**, n males (%)	6,911 (34.4%)	6,916 (34.5%)	0.96
**Body mass index** (kg/m^2), n (%)			0.12
<20.0	76 (0.4%)	87 (0.4%)	
≥20.0 to <25.0	1,185 (5.9%)	1,260 (6.3%)	
≥25.0 to <30.0	3,563 (17.8%)	3,641 (18.2%)	
≥30.0 to <35.0	3,015 (15.0%)	3,040 (15.2%)	
≥35.0 to <40.0	1,110 (5.5%)	1,171 (5.8%)	
≥40.0	392 (2.0%)	420 (2.1%)	
Not available	10,723 (53.4%)	10,445 (52.1%)	
**Follow-up** (months), mean (SD)	80.7 (43.6)	81.4 (41.3)	0.10
**Comorbidities, n (%)**			
CKD	2,045 (10.2%)	1,971 (9.8%)	0.22
Cardiac and/or valvular diseases[a]	7,184 (35.8%)	7,120 (35.5%)	0.50
Diabetes	6,195 (30.9%)	6,202 (30.9%)	0.94
Hypertension	16,928 (84.4%)	17,110 (85.3%)	0.01

sCr (mg/dL), mean (SD)	1.02 (0.41)	1.01 (0.35)	0.08
eGFR (ml/min/1.73m²), mean (SD)	65.6 (22.7)	66.7 (22.0)	0.001
eGFR KDIGO categories[ab]			<0.0001
Grade 1 (≥90 ml/min/1.73m²)	1,021 (12.0%)	1,223 (13.2%)	
Grade 2 (≥60 to <90 ml/min/1.73m²)	3,979 (46.7%)	4,349 (46.8%)	
Grade 3 (≥30 to <60 ml/min/1.73m²)	3,098 (36.4%)	3,404 (36.6%)	
Grade 4 (≥15 to <30 ml/min/1.73m²)	378 (4.4%)	291 (3.1%)	
Grade 5 (<15 ml/min/1.73m²)	39 (0.5%)	25 (0.3%)	
**Drug exposure in the 12 months prior to the index date,** n (%)			
α-blockers	742 (3.7%)	829 (4.1%)	0.03
β-blockers	3,636 (18.1%)	3,725 (18.6%)	0.25
Calcium channel blockers	5,482 (27.3%)	5,557 (27.7%)	0.40
Oral antiplatelet agents	5,679 (28.3%)	5,819 (29.0%)	0.12
Anticoagulants	3,176 (15.8%)	3,025 (15.1%)	0.04
Digoxin	589 (2.9%)	611 (3.1%)	0.52
Antianginal and antiarrhythmic drugs	2,372 (11.8%)	2,431 (12.1%)	0.36
Statins	8,001 (39.9%)	8,219 (41.0%)	0.03
Antidiabetic drugs	4,755 (23.7%)	4,820 (24.0%)	0.45
Acyclovir	29 (0.1%)	34 (0.2%)	0.53
Beta-lactams	2,661 (13.3%)	2,776 (13.8%)	0.09
Quinolones	1,687 (8.4%)	1,796 (9.0%)	0.05
Sulfonamides	107 (0.5%)	99 (0.5%)	0.58
Rifampicin	17 (0.1%)	10 (0.1%)	0.18
Immunosuppressants	90 (0.5%)	80 (0.4%)	0.44
Acetaminophen	8,831 (44.0%)	8,644 (43.1%)	0.06
Systemic corticosteroids	1,365 (6.8%)	1,334 (6.7%)	0.54
Bisphosphonates	1,019 (5.1%)	1,025 (5.1%)	0.89
Allopurinol	1,771 (8.8%)	1,821 (9.1%)	0.38
Penicillamin	0 (0.0%)	0 (0.0%)	1.00
Lithium	7 (0.03%)	11 (0.05%)	0.35
Nitrogen mustards	3 (0.01%)	0 (0.0%)	0.25
Quinolines	38 (0.2%)	45 (0.2%)	0.44
Gold preparations	0 (0.0%)	0 (0.0%)	1.00
**Length of continuous TW exposure [c] (months),** mean (SD)	4.7 (3.8)	3.9 (3.6)	<0.0001
**Length of overall TW exposure (months),** mean (SD)	5.0 (3.8)	4.2 (3.6)	<0.0001

Chronic kidney disease: chronic kidney disease; eGFR: estimated glomerular filtration rate; sCr: serum creatinine

a. Heart failure, ischemic cardiopathy (including acute coronary syndrome, acute myocardial infarction, angor pectoris), cardiac valvulopathy, arrhythmia, atrial fibrillation and/or auricular flutter, congenital cardiovascular disorders.

b. Available for n=12,136 from the group with TW including metamizole, and n=16,448 from the group with TW including a NSAIDS.

c. Continuous TW: when there was a gap of no more than 30 days between the last day of a TW exposure and the first day of a subsequent TW exposure.

Table S4. Risk of hospitalisation due to acute kidney injury (AKI) after propensity score matching

	Cases, n (%)	Controls, n (%)	RR (95%CI)	P
TW including metamizole and not NSAID	3,013 (53.7%)	17,051 (49.4%)	1 (Ref.)	
TW including NSAID and not metamizole	2,603 (46.4%)	17,461 (50.6%)	0.87 (0.80, 0.96)	0.004

NSAID: non-steroidal anti-inflammatory drug; RR: Relative Risk; TW: triple whammy; 95%CI: 95% confidence

Table S5. Risk of hospitalisation due to acute kidney injury (AKI) according to exposure time.

		Cases, n (%)	Controls, n (%)	OR (95%CI)	Adjusted [a] OR (95%CI)
TW including metamizole and not NSAID	1 month	1,006 (11.9%)	5,349 (9.4%)	1 (ref.)	1 (ref.)
	2-6 months	1,657 (19.7%)	8,472 (15.0%)	1.09 (0.96, 1.23)	1.05 (0.91, 1.22)
	7-12 months	1,460 (17.3%)	7,420 (13.1%)	1.10 (0.97, 1.25)	1.20 (1.04, 1.39)
TW including NSAID and not metamizole	1 month	1,421 (16.9%)	12,280 (21.7%)	0.58 (0.51, 0.65)	0.77 (0.67, 0.89)
	2-6 months	1,678 (19.9%)	14,040 (24.8%)	0.62 (0.55, 0.70)	0.84 (0.73, 0.97)
	7-12 months	1,200 (14.3%)	9,094 (16.1%)	0.69 (0.61, 0.79)	1.08 (0.93, 1.26)

NSAID: non-steroidal anti-inflammatory drug; OR: Odds ratio; TW: triple whammy; 95%CI: 95% confidence
aAdjusted for comorbidities registered up to the index date (chronic kidney disease, cardiovascular and/or valvular disease, diabetes, hypertension, cerebrovascular diseases, chronic liver disease, chronic obstructive pulmonary disease, alcohol dependence, smoking, substance abuse), body mass index category, and exposure to the following drugs in the 12 months prior to the index date: α-blockers, β-blockers, calcium channel blockers, oral antiplatelet agents, anticoagulants, digoxin, antianginal, antiarrhythmic drugs, statins, antidiabetic drugs, acyclovir, beta-lactams, quinolones, sulfonamides, rifampicin, immunosuppressants, acetaminophen, systemic corticosteroids, bisphosphonates, allopurinol.

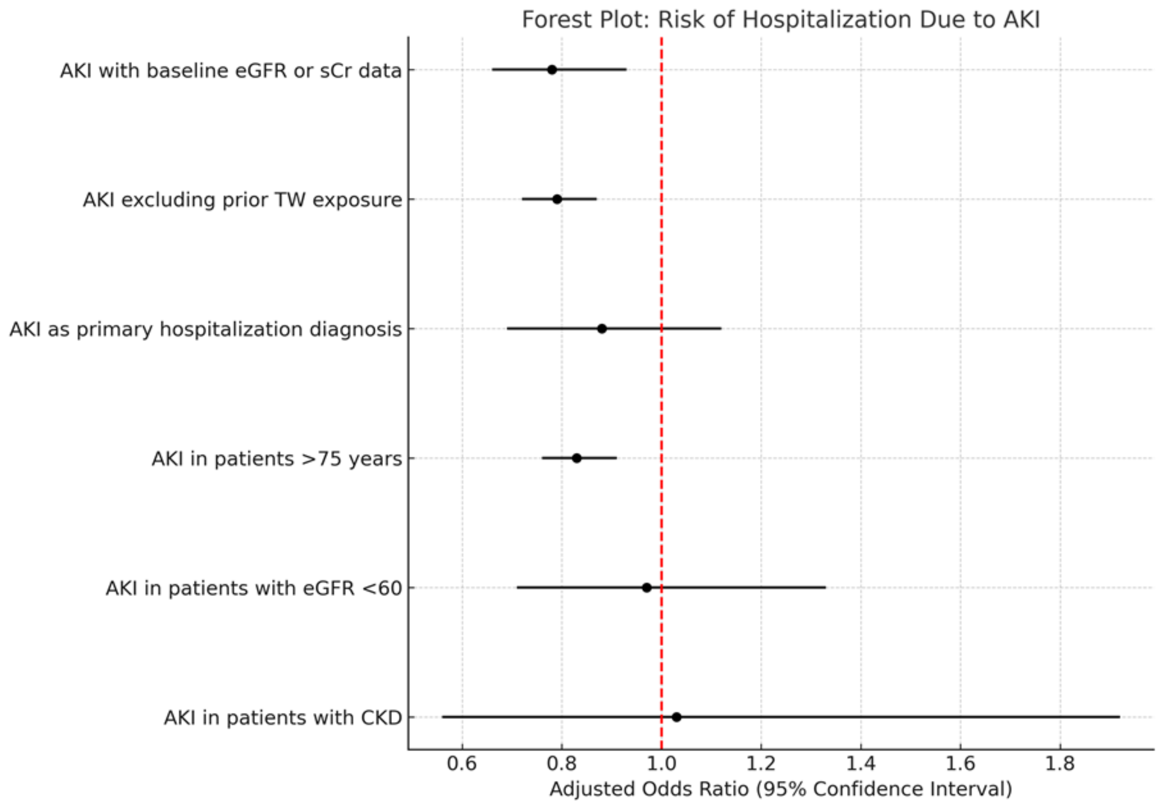

"Figure S1. Forest plot for sensitivity and subgroup analyses of acute kidney injury (AKI)

Table S6. Sensitivity and subgroup analysis

Analysis	Adjusted [a] OR	95 %, CI
AKI with baseline eGFR or sCr data	0.78	0.66-0.93
AKI excluding prior TW with metamizole/NSAID in sequential exposure	0.79	0.72-0.87
AKI as primary hospitalization diagnosis	0.88	0.69-1.12
AKI in patients >75 years	0.83	0.76-0.91
AKI in patients with eGFR <60 mL/min/1.73m2 at baseline	0.97	0.71-1.33
AKI in patients with CKD	1.03	0.56-1.92

NSAID: non-steroidal anti-inflammatory drug; OR: Odds ratio; TW: triple whammy; 95%CI: 95% confidence aAdjusted for comorbidities registered up to the index date (chronic kidney disease, cardiovascular and/or valvular disease, diabetes, hypertension, cerebrovascular diseases, chronic liver disease, chronic obstructive pulmonary disease, alcohol dependence, smoking, substance abuse), body mass index category, and exposure to the following drugs in the 12 months prior to the index date: α-blockers, β-blockers, calcium channel blockers, oral antiplatelet agents, anticoagulants, digoxin, antianginal, antiarrhythmic drugs, statins, antidiabetic drugs, acyclovir, beta-lactams, quinolones, sulfonamides, rifampicin, immunosuppressants, acetaminophen, systemic corticosteroids, bisphosphonates, allopurinol.

Table S7. Risk of hospitalisation due to AKI restricting to cases and controls with eGFR or sCr data at baseline (at the beginning or in the 12 months previous to the beginning of the follow-up) (sensitivity analyses) with multiple imputations

	Cases (imputed) No	Controls (imputed) No	OR (imputed data) (crude)	P value	Adjusted OR [a] (imputed data)	P value
TW including metamizole and not NSAID	52.6%	43.4%	1 (ref.)		1 (ref.)	
TW including NSAID and not metamizole	47.4%	56.6%	0.62 (0.54-0.71)	<0.0001	0.83 (0.70, 0.98)	0.03

NSAID: non-steroidal anti-inflammatory drug; OR: Odds ratio; TW: triple whammy; 95%CI: 95% confidence aAdjusted for comorbidities registered up to the index date (chronic kidney disease, cardiovascular and/or valvular disease, diabetes, hypertension, cerebrovascular diseases, chronic liver disease, chronic obstructive pulmonary disease, alcohol dependence, smoking, substance abuse), body mass index category, and exposure to the following drugs in the 12 months prior to the index date: α-blockers, β-blockers, calcium channel blockers, oral antiplatelet agents, anticoagulants, digoxin, antianginal, antiarrhythmic drugs, statins, antidiabetic drugs, acyclovir, beta-lactams, quinolones, sulfonamides, rifampicin, immunosuppressants, acetaminophen, systemic corticosteroids, bisphosphonates, allopurinol.
